# Delayed mid-sleep time associated with weight gain while controlling for eating behaviors and ADHD symptoms during the COVID-19 pandemic

**DOI:** 10.1192/j.eurpsy.2023.375

**Published:** 2023-07-19

**Authors:** A. Kandeğer, Ö. F. Uygur, E. Yavuz, Y. Selvi

**Affiliations:** ^1^Psychiatry, Selcuk University, Konya; ^2^Psychiatry, Atatürk University, Erzurum, Türkiye

## Abstract

**Introduction:**

Society’s sleep-wake cycle and eating behaviors have altered as the psychological outcomes of the COVID-19 pandemic.

**Objectives:**

The aim is to examine the relationship between sleep-wake rhythms, eating behaviors (dieting, oral control, and bulimic behaviors) and ADHD symptoms with weight gain during the COVID-19 pandemic.

**Methods:**

Participants were 578 female university students divided into three groups based on weight change during COVID-19 who lost weight (WL), those whose weight did not change (nWC), and who gained weight (WG). They completed an online survey including, a consent form regarding voluntary participation, the socio-demographic form in which requested information about weight change in the last year, the Pittsburg Sleep Quality Index (PSQI), Eating Attitudes Test, Adult ADHD Severity Rating Scale, Wender Utah Rating Scale. The study was approved by the Selçuk University Local Ethics Committee (Decision Number: 2021/369).

**Results:**

The sleep-wake phase was more delayed in WGs compared to the other two groups. The bulimic behavior score was higher and the oral control behavior score was lower in the WG group than in the nWC group. In the first step of the hierarchical regression analysis to determine factors associated with weight change, childhood and current ADHD symptoms did not show an association with weight change. In the second step, sleep-wake parameters were added to the analysis, and mid-sleep time was a strong predictor of weight gain (β= 4.71, t= 2.18, p = 0.03). In the third step, in which disordered eating behaviors were added to the analysis, bulimic behaviors (β= 0.20, t= 3.20, p= 0.001) were associated with weight gain and oral control behaviors (β= -0.11, t= -3.24, p= 0.001) were associated with weight loss.

**Conclusions:**

WGs had a delayed sleep phase more than nWCs and WLs in the one-year period during the COVID 19 outbreak. Chronotherapeutic approaches that regulate sleep-wake rhythm may facilitate weight control of individuals during stressful periods such as COVID-19 outbreak.

**Disclosure of Interest:**

None Declared

